# Genomic Hotspots for Adaptation: The Population Genetics of Müllerian Mimicry in *Heliconius erato*

**DOI:** 10.1371/journal.pgen.1000796

**Published:** 2010-02-05

**Authors:** Brian A. Counterman, Felix Araujo-Perez, Heather M. Hines, Simon W. Baxter, Clay M. Morrison, Daniel P. Lindstrom, Riccardo Papa, Laura Ferguson, Mathieu Joron, Richard H. ffrench-Constant, Christopher P. Smith, Dahlia M. Nielsen, Rui Chen, Chris D. Jiggins, Robert D. Reed, Georg Halder, Jim Mallet, W. Owen McMillan

**Affiliations:** 1 Department of Genetics, North Carolina State University, Raleigh, North Carolina, United States of America; 2 Department of Biology, University of Puerto Rico–Rio Piedras, San Juan, Puerto Rico; 3 Department of Zoology, University of Cambridge, Cambridge, United Kingdom; 4 Department of Biochemistry and Molecular Biology, M. D. Anderson Cancer Center, University of Texas, Houston, Texas, United States of America; 5 Department of Ecology and Evolutionary Biology, University of California Irvine, Irvine, California, United States of America; 6 CNRS UMR 7205, Département Systématique et Evolution, Muséum National d'Histoire Naturelle, Paris, France; 7 School of Biosciences, University of Exeter in Cornwall, Pernyn, United Kingdom; 8 Bioinformatic Resource Center, North Carolina State University, Raleigh, North Carolina, United States of America; 9 Baylor Human Genome Sequencing Center, Houston, Texas, United States of America; 10 Galton Laboratory, University College London, London, United Kingdom; University of Arizona, United States of America

## Abstract

Wing pattern evolution in *Heliconius* butterflies provides some of the most striking examples of adaptation by natural selection. The genes controlling pattern variation are classic examples of Mendelian loci of large effect, where allelic variation causes large and discrete phenotypic changes and is responsible for both *convergent* and *highly divergent* wing pattern evolution across the genus. We characterize nucleotide variation, genotype-by-phenotype associations, linkage disequilibrium (LD), and candidate gene expression patterns across two unlinked genomic intervals that control yellow and red wing pattern variation among mimetic forms of *Heliconius erato*. Despite very strong natural selection on color pattern, we see neither a strong reduction in genetic diversity nor evidence for extended LD across either patterning interval. This observation highlights the extent that recombination can erase the signature of selection in natural populations and is consistent with the hypothesis that either the adaptive radiation or the alleles controlling it are quite old. However, across both patterning intervals we identified SNPs clustered in several coding regions that were strongly associated with color pattern phenotype. Interestingly, coding regions with associated SNPs were widely separated, suggesting that color pattern alleles may be composed of multiple functional sites, conforming to previous descriptions of these loci as “supergenes.” Examination of gene expression levels of genes flanking these regions in both *H. erato* and its co-mimic, *H. melpomene*, implicate a gene with high sequence similarity to a kinesin as playing a key role in modulating pattern and provides convincing evidence for parallel changes in gene regulation across co-mimetic lineages. The complex genetic architecture at these color pattern loci stands in marked contrast to the single casual mutations often identified in genetic studies of adaptation, but may be more indicative of the type of genetic changes responsible for much of the adaptive variation found in natural populations.

## Author Summary

Identifying the genetic changes responsible for beneficial variation is essential for understanding how organisms adapt. Here, we use a combination of mapping, population genetic analysis, and gene expression studies to identify the genomic regions responsible for phenotypic evolution in the Neotropical butterfly *Heliconius erato*. *H. erato*, together with its co-mimic *H. melpomene*, have undergone parallel and concordant radiations in their warningly colored wing patterns across Central and South America. The “genes” underlying the *H. erato* color pattern radiation are classic examples of Mendelian loci of large effect and are under strong natural selection. Nonetheless, we do not see a clear molecular signal of recent natural selection, suggesting that the *H. erato* color pattern radiation, or the alleles that underlie it, may be quite old. Moreover, rather than being single locus, the genetic patterns suggest that multiple, widely dispersed loci may underlie pattern variation in *H. erato*. One of these loci, a kinesin gene, shows parallel expression differences between races during wing pattern formation in both *H. erato* and *H. melpomene*, suggesting that it plays an important role in pattern variation. High rates of recombination within naturally occurring *H. erato* hybrid zones mean that finer genetic dissection will allow us to localize causative sites and better understand the history and molecular basis of this extraordinary adaptive radiation.

## Introduction

Understanding how adaptive phenotypes arise is vital for understanding the origins of biodiversity and for predicting how organisms will respond to novel selective pressures [1]. Nonetheless, there are still only a handful of examples where the molecular elements underlying adaptive variation in nature have been identified [2]–[6]. This situation is changing as new technologies make it possible to leverage nature's diversity and focus research directly on taxa that are both ecologically tractable and possess characteristics (life history switches, behavioral modifications, or phenotypic differences) with *a priori* evidence of their adaptive role [7]–[10]. The data that will emerge from these studies promise fresh insights into the genetic architecture and origins of functional variation and an exciting new understanding of the interplay between genes, development, and natural selection.

*Heliconius* butterflies offer exceptional opportunities for genomic level studies designed to understand how adaptive morphological diversity is generated in nature [11]–[13]. The group is renowned as one of the great insect radiations and provides textbook examples of adaptation by natural selection, mimicry, and speciation [14],[15]. The vivid wing patterns of *Heliconius* are adaptations that warn potential predators of the butterflies' unpalatability and also play a key role in speciation [16]–[18]. Perhaps the greatest strength of *Heliconius* for understanding the origins of functional variation lies is the wealth of parallel and convergent adaptation in the group- a pattern best exemplified by the parallel mimetic radiations of *H. erato* and *H. melpomene* [19]–[23]. The two species are distantly related and never hybridize [24],[25]; yet, they possess nearly identical wing patterns and have undergone nearly perfectly congruent radiations into over 25 distinctively different color pattern races [21]. The convergent and divergent color pattern changes within and between *Heliconius* species provide “natural” replicates of the evolutionary process where independent lineages have produced similar phenotypes due to natural selection. Indeed, within both the *H. erato* and *H. melpomene* radiations, there are multiple disjunct populations that share identical, yet possibly independently evolved, wing patterns [26],[27] (for an alternative, shifting balance view, see [22],[28]). Moreover, recent comparative research has demonstrated that the diversity of color patterns found within *H. erato*, *H. melpomene* and in other *Heliconius* species, is modulated by a small number of apparently homologous genomic intervals [29]–[31], which provides a powerful evolutionary framework for examining the origins of functional variation and allows insights into the repeatability of evolution.

**Figure 1:**
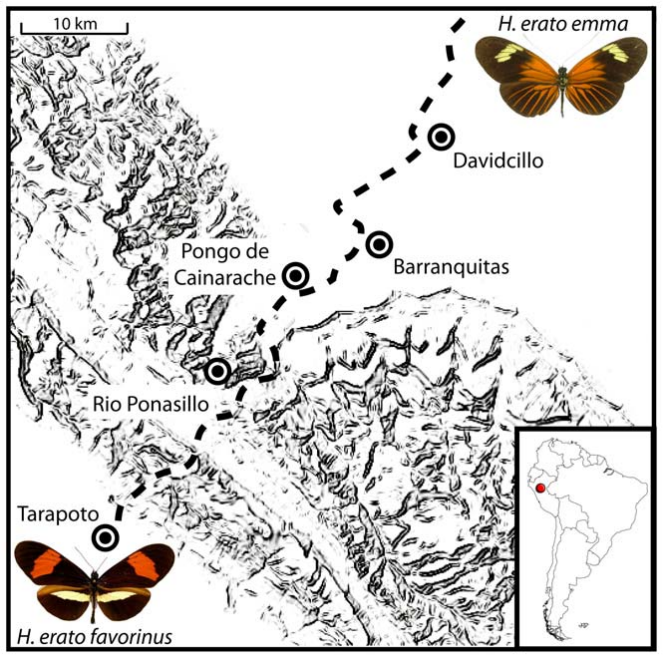
Sampling sites across the transition between *H. e. favorinus* and *H. e. emma*. Geographic representation of the five locations where *H. erato* was sampled across the Eastern Peruvian hybrid zone. Dotted line is approximate location of the Tarapoto-Yurimaguas road that transects the hybrid zone and was used for sampling. The *D* locus affects the presence of the proximal red patch (“dennis”), red hindwing rays and the forewing band color. The *Cr* locus is responsible for the presence of the hindwing yellow bar and interacts with the *Sd* locus to affect the shape of the forewing band and hindwing bar.

The patchwork of differently patterned races in *H. erato* and *H. melpomene* is stitched together by dozens of narrow hybrid zones [20]–[22], allowing detailed analysis of the forces that generate and maintain adaptive variation in this group [32]. Here, and in our companion paper [33], we exploit concordant hybrid zones to explore patterns of nucleotide diversity and linkage disequilibrium (LD) across two of the three interacting genomic regions that control most of the adaptive differences in wing color patterns. The transition between the “postman”, *H. e. favorinus* and *H. m. amaryllis*, and “rayed”, *H. e. emma* and *H. m. agalope*, races of the two co-mimics in eastern Peru is one of the best described hybrid zones in *Heliconius* and occurs over a distance of slightly more than 10 km (Figure 1 and [33]). Strong natural selection maintains this sharp phenotypic boundary in both species and per locus selective coefficients on color pattern loci are estimated to be greater than 0.2 both using field release experiments and by fitting the observed cline in allelic frequencies at each of the color pattern loci to a theoretical cline maintained by frequency dependent selection [34],[35]. Despite strong natural selection, there are no strong pre- or post-mating barriers to hybridization between races of either *H. erato* or *H. melpomene* and in the center of the hybrid zone there is frequent admixture between divergent color pattern races.

Our study focuses on two *H. erato* patterning loci, *D* and *Cr*. These two loci map to different linkage groups and interact to control major differences in the wing color patterns of *H. erato* races. The chromosomal regions tightly linked with the *D* and *Cr* loci in *H. erato* were recently identified [36]–[38] and map to homologous regions of the genome that control similar color pattern changes in *H. erato*'s co-mimic, *H. melpomene* [29],[31]. Variation in *D* in *H. erato* and *D/B* in *H. melpomene* cause analogous changes in the distribution of red pigments on the fore- and hindwings (see[30],[31],[39]). Similarly, *Cr* (*H. erato*) and the *Yb*-complex (*H. melpomene*) cause similar shifts in the distribution of melanic scales revealing underlying white and yellow pattern elements (see [29]). This region also contains the *H. numata P* locus, a close relative of *H. melpomene*. However, the *P* locus causes dramatically different pattern changes among sympatric races of *H. numata* highlighting the extraordinary ‘jack-of-all-trades’ flexibility of these genomic regions [29].

Wing pattern variation across *Heliconius* hybrid zones serves as a “natural” laboratory for genome level research into processes that generate and maintain adaptive variation. One of the most extensively studied *Heliconius* hybrid zones is found in Eastern Peru, where Mallet and coworkers estimated the strength of natural selection on the three unlinked color pattern loci that control phenotypic differences between “rayed” and “postman” races of *H. erato* [34],[35],[40]. We have taken the next step and used this same Peruvian *H. erato* hybrid zone to make four major advancements: (1) we have identified and sequenced narrow genomic intervals containing two of the three interacting loci that cause major adaptive shifts in wing patterns; (2) we have documented a rapid decay of LD in natural populations across a sharp phenotypic transition both within genes and across these intervals; (3) we have identified several genes strongly associated with the transition in warningly-colored wing patterns; and (4) we have examined expression levels in these and adjacent genes during wing development. These data, in combination with data presented in the companion paper [33], refine our understanding of the molecular nature of color pattern loci and suggest that multiple functional sites underlie adaptive morphological variation in *Heliconius*.

## Results

### Fine mapping and sequencing of color pattern intervals in *H. erato*

**Figure 2:**
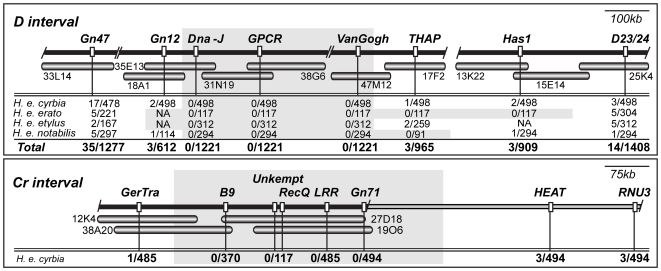
BAC tile paths and fine mapping across the *D* and *Cr* color pattern intervals. Individual BAC clones tiling across the color pattern intervals are represented by horizontal shaded bars, with clone name provided directly below. Black horizontal bar above BAC tile path represents consensus sequence assembled from overlapping BACs. Slashes indicate gaps in the consensus sequence across the interval. There were two small gaps (≈10kb between *H. erato* clone 33L14 and 18A1 and ≈5kb between 38G06 and 47M12) and one large gap (≈250 kb) in our assembly based on comparisons to *Bombyx mori* and *H. melpomene*. For the *Cr* interval, the grey horizontal bar extending to the right of the black horizontal bar represents a region with no available information on recombination. Vertical white markers denote approximate positions of genetic markers used for brood mapping, with marker names stated directly above. Below each marker is the number of individuals showing a recombination event between the genetic marker and color pattern phenotype over the total number of individuals genotyped. For the *D* locus, four phenotypically distinct races of *H. erato* were used for fine mapping in crosses with *H. himera* [39],[100],and the results for each race are provided separately. Genetic markers designated NA were either not polymorphic or could not be reliably scored in the corresponding crosses. Shaded areas denote approximate locations of ‘zero recombinant intervals’.

Building on earlier work, including the initial BAC tile path of *H. melpomene D/B* locus [31], we sequenced 10 *H. erato* BACs representing over 1 Mb of genomic sequence around the *D* locus (Figure 2). Across the *D* BAC tile path, we surveyed over 1200 individuals from our *H. erato* x *H. himera* F_2_ and backcross mapping families at several molecular markers, and identified an approximately 380 kb interval between the markers *Gn12* and *THAP* that had no recombination events between color pattern phenotype and genotype (shaded region on Figure 2). The lack of recombinants across this zero recombinant window stood in marked contrast to the pattern observed at both the 5′- and 3′-end of our tile path. At both ends of the region, the number of individuals showing a recombinant event between a genetic marker and color pattern phenotype was similar to the expected 276 kb/cM based on previous mapping work [39], but then dropped off rapidly in the centre of the region. The drop off was particularly marked on the 5′end of the interval, where the number of recombinant events fell from 35 individuals at *GN47* to 0 individuals at *Dna-J* over a span of approximately 200 kb.

We also identified the genomic interval containing the *Cr* locus, although in this case, we do not yet have a BAC tile path across the entire interval. The 5′-end of *Cr* interval is marked by the locus *GerTra*, where we identified a single recombinant among nearly 500 *H. erato cyrbia* x *H. himera* F_2_ and backcross individuals. At the 3′-end, we observed 3 *Cr* recombinants at *HEAT*, which is about 600 kb from *GerTra* based on comparisons to the *Bombyx mori* genome (Figure 2). We sequenced three new BAC clones yielding approximately 420kb of sequence at the 5′-end of the *Cr* interval. Across our physical sequence of the *Cr* interval, we found no recombinant individuals at markers 3′ of *GerTra (B9*, *recQ*, *Invertase*, *LRR*, *and GN 71)* a span of approximately 340 kb (Figure 2). Thus, as with the *D* locus interval, there were fewer recombination events than expected based on previous estimates of the relationship between physical and recombination distance.

### Genetic diversity and LD across color pattern intervals

We estimated genetic diversity from 76 individuals collected from five locations along a 30 km transect, representing three distinct populations, phenotypically pure *H. e. favorinus* (n = 20), admixed individuals (n = 42), and largely pure *H. e. emma* (n = 14) (Figure 1). In total, we assayed variation across 12,660 bp from 25 coding regions including 13 regions from the *D* interval, 10 from the *Cr* interval, and 3 unlinked to each other or any color pattern locus in *H. erato* (Table S1). There were 1542 polymorphic sites among the sampled individuals. Most of these (1110) positions had minor allele frequencies of less than 5 percent. Of the remaining 432 polymorphic sites, ten had more than two variant bases.

The mean nucleotide diversity (*π*, average number of pair-wise differences between sequences) among all sampled gene regions in *H. erato* was 0.022±0.017. In general, there were no strong differences in nucleotide diversity among loci tightly linked to color pattern genes relative to loci unlinked to color pattern (Table 1). Nucleotide diversity was also very similar among the three sampled *H. erato* populations, except for a few gene regions at the *Cr* locus in the admixed population that showed slightly elevated estimates of nucleotide diversity (Table 1). Over half the coding regions sampled in this study had patterns of nucleotide diversity not consistent with simulations of neutral evolution, in at least one of the three populations sampled. Near the *D* locus, many coding regions had negative Tajima's *D* values that were significantly different from neutral expectation (Table 1). However, there seemed to be little pattern to these departures from neutrality. For example, the coding regions at the *D* locus most strongly associated with color pattern variation (see below) all showed patterns consistent with the neutral model. In contrast, at the *Cr* locus, the two coding regions with associated SNPs accounted for about half of the significant deviations from neutrality in genes across this region (Table S1). We also observed significant deviations from neutrality at loci unlinked to color pattern variation. In particular, the *Heliconius wingless* homologue deviated in all three populations examined (Table S1). Overall nucleotide diversity was generally greater in the *H. erato* (mean *π* = 0.022±0.017) than in *H. melpomene* (mean *π* = 0.012±0.019, [33]) but the differences were much less than previously reported for nuclear introns [27]. Moreover, in *H .melpomene*, as in *H. erato*, there were no striking differences in diversity between loci within and outside of color pattern intervals, nor consistent departures from neutrality within color pattern intervals.

**Table 1:** Estimates of genetic diversity across the *H. erato* hybrid zone.

*D* locus		*Cyta*	*Dna-J*	*3P*	*Slu7*	*Kinesin*	*GPCR*	*Abh*	*VanGogh*	*Gn6*	*THAP*	*Gn18*	*Has1*
Position along interval		91,726	165,009	237,736	283,611	299,335	309,248	322,422	513,028	517,433	552,580	579,264	1,442,402
π	favorinus	0.0034	0.0127	0.0916	0.0084	0.0073	0.0082	0.0078	0.0075	0.0036	0.0063	0.0224	0.0046
	admix	0.0031	0.0178	0.0792	0.01	0.0132	0.0165	0.0207	0.0129	0.025	0.0115	0.0305	0.01
	emma	0.0037	0.0158	0.0598	0.011	0.0101	0.0257	0.0295	0.0141	0.0245	0.011	0.0478	0.0142
Tajima's D	favorinus	2.839*	−0.98	−2.202*	−1.628	−0.359	−1.241	−0.707	−1.309	−1.954*	−0.674	−1.448	−1.017
	admix	2.709*	−1.396	−2.056*	−1.721	−2.052	−0.929	−0.558	−0.609	−1.791*	−1.682	−1.599	−1.850*
	emma	2.237*	−1.705*	−2.493**	−1.901	−1.776	−1.439	−0.26	−0.127	−1.699	−1.792*	−2.560*	−2.483*
*Cr* locus		*Forkhead*	*B9*	*Treh(A)*	*Treh(B)*	*BESS*	*WD40*	*Unkempt*	*recQ*	*Invertase*	*LRR*		
Position along interval		1	54,672	66,809	67,583	104,621	130,877	177,423	190,761	201,977	249,391		
π	favorinus	0.0222	0.0252	0.0091	0.0282	0.0241	0.0256	0.03825*	0.0078	0.019	0.0153		
	admix	0.0242	0.0307	0.0121	0.0508	0.0357	0.0276	0.0803	0.0283	0.037	0.0258		
	emma	0.0402	0.0193	0.0082	0.0246	0.0414	0.0295	0.15611*	0.0069	0.0216	0.0211		
Tajima's D	favorinus	−1.535	−1.436	−0.671	−2.136*	−0.833	−0.162	−2.518**	−1.983*	−0.563	−1.675*		
	admix	−1.546	−1.568	−1.088	−2.447*	−1.314	−1.102	−1.874*	−2.209*	−1.701	−0.868		
	emma	−2.103*	−1.061	−0.136	−1.691	−1.378	−0.926	−3.132**	−1.842*	−0.802	−0.862		
Unlinked loci		*Caspase*	*SUZ12*	*Wingless*									
π	favorinus	0.0148	0.0126	0.0228									
	admix	0.0142	0.0173	0.0382									
	emma	0.0136	0.0163	0.0194									

**Figure 3:**
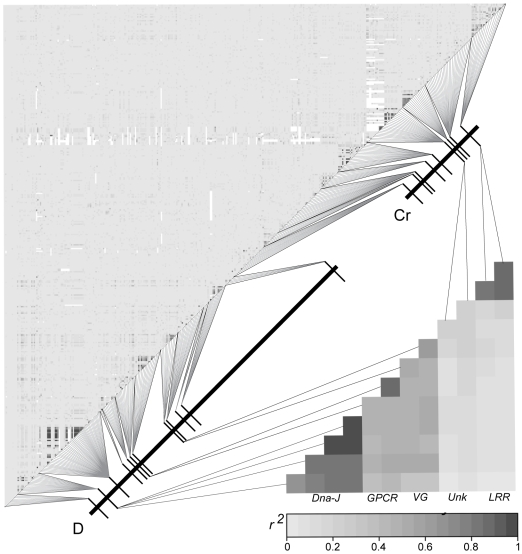
Lack of LD between SNPs across the *D* and *Cr* intervals in the Peruvian hybrid zone. Correlation matrix of composite LD estimates among SNPs from the 22 coding regions sampled across the *D* and *Cr* intervals using all 76 individuals. SNPs are concatenated by their position along the *D* and *Cr* intervals. The upper left matrix shows LD between the 401 SNPs sampled across the *D* and *Cr* intervals. The lower right matrix only shows SNPs from the *D* and *Cr* intervals that are strongly associated with wing color pattern.

Linkage disequilibrium among SNPs decayed precipitously with physical distance across both the *D* and *Cr* intervals (Figure 3 and Figure S2). This observation was true for phenotypically pure populations collected at either side of the sharp phenotypic transition (Figure S1), for “admixed” populations in the center of the transition zone (Figure S1), and even for the population as a whole (Figure 3). The only sites with high estimates of *r^2^* (>0.5) were found within the same coding regions. All other estimates of *r^2^* were near zero (Figure 3), including values between *D* and *Cr* interval SNPs (Figure 3). The lack of strong LD in populations across this phenotypic boundary was perhaps best exemplified by the LD patterns within loci - for all loci, including those that fell within our zero recombinant windows, short-range LD decayed to *r^2^* values near zero within 300–500 bp. Although broadly similar, the pattern of LD differed from what was observed in *H. melpomene* (see [33] Figure S2), where LD generally extended farther and there was some evidence for significant haplotype structure and long-distance LD among sites.

### Genotype-by-phenotype associations

#### *D* locus associations

**Figure 4:**
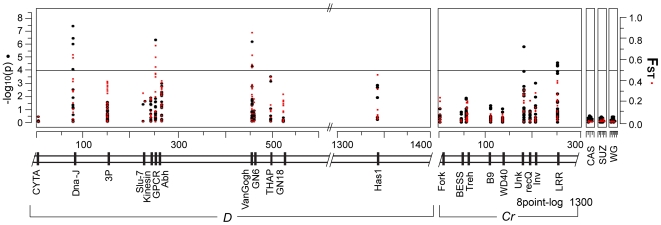
Several sites in multiple coding regions are associated with the transition in *D* and *Cr* color patterns. Plot of genotype-phenotype associations (black circles) and population differentiation (red squares) across the *D*, *Cr* and unlinked intervals. The left axis is the strength of associations (log_10_ of the probability of a genotype-by-phenotype association) between genotypes and color patterns. The right axis measures degree of population differentiation, measured as *F_ST_*, between *H. e. favorinus* and *H. e. emma*. Distance across the genomic intervals is in kilobases. Points above the horizontal show a significant genotype-by-phenotype association using a bonferroni correction to adjust for multiple tests (*α* = 0.05, *n* = 432).

Within the backdrop of the rapid decay of LD, we identified strong genotype-by-phenotype associations at a number of positions across both the *D* and *Cr* intervals. Although there were no fixed differences between races, we identified strong associations between SNP variants and the *D* phenotype in three coding regions, we termed *Dna-J*, *GPCR*, and *VanGogh* (Figure 4). The three coding regions fell within a ∼380 kb interval that correlated perfectly with the zero recombinant window identified in our linkage analysis (see Figure 2). Each of the regions had between 2–5 significantly associated sites as well as SNPs that showed no association interspersed across the coding regions (see Table S2 for complete list of the genotype-by-phenotype associations). The associations at each of these three coding regions was primarily driven by nucleotides that were nearly fixed in individuals homozygous for the *H. e. emma D* phenotype. The strongest associations were among SNPs at *Dna-J*, including three synonymous substitutions and two non-synonymous substitutions that resulted in an isoleucine/valine polymorphism at positions 73,699 and 73,753. In both cases, valine was strongly associated with *H. e. emma D* color pattern. At *GPCR* there were two synonymous substitutions strongly associated with *D* phenotype. At *VanGogh* there was one synonymous substitution and one non-synonymous substitution strongly associated with the *D* phenotype.

In general, estimated levels of differentiation among populations were very similar to the association results- loci that had strongly associated sites also had high *F_ST_* values. These patterns of genotype-by-phenotype association and population differentiation stand in marked contrast to observations at unlinked loci and loci that fell outside the zero recombinant window. The average *F_ST_* between the pure *H. e. favorinus* and pure *H. e. emma* populations was over 2-fold greater for the coding regions strongly associated with the *D* phenotype (0.34), relative to the other coding regions within the *D* zero recombinant window that did not show significant associations (0.16, see Figure 2 and Table S2). Outside the zero recombinant window, levels of population differentiation were lower than inside, but remained higher than levels observed in unlinked loci (Figure 4 and Table S2).

#### *Cr* locus associations

The strength of associations and estimates of population differentiation were lower across the *Cr* interval relative to the *D* interval. Only two of the 9 genes sampled contained SNPs significantly associated with the *Cr* phenotype: one gene being a coding region with high sequence similarity to the *Drosophila* transcription factor *Unkempt* and the other gene being a coding region with a leucine-rich (*LRR*) protein motif. These two regions were separated by approximately 80 kb and, similar to the pattern in the *D* interval, were separated from each other by loci that contained no SNPs associated with color pattern (Figure 4). Also similar to the *D* locus, associated sites in the same gene were often interspersed by SNPs that showed no association. Three out of the four strongly associated SNPs across the *Cr* pattern intervals were non-synonymous substitutions. Across the Cr interval the average *F_ST_* among sampled coding regions between the two phenotypically pure populations was 0.035, or approximately 8 times lower than the average *F_ST_* across the *D* interval (Table 2). Even the two loci that contained sites significantly associated with color pattern phenotype showed only a moderate degree of population differentiation (average *F_ST_* = 0.145 for *LRR* and average *F_ST_* = 0.021 for *Unkempt*) between the phenotypically pure populations sampled in this study (Figure 4, Table S2).

**Table 2:** High genetic differentiation near color pattern loci.

	*D* linked loci Mean *F_ST_*	*Cr* linked loci Mean *F_ST_*	Unlinked loci Mean *F_ST_*
*H. e. favorinus vs. H .e. emma*	0.216±0.111 (n = 12)	0.03±0.045 (n = 10)	0.007±0.015 (n = 3)

#### LD between associated SNPs

In general, associated SNPs within each color pattern interval were in higher LD than unassociated sites, but they showed a similar rapid decay with distance (see Figure 3 and Figure S2). Thus, while LD between associated SNPs in the same coding regions could be strong, LD between associated SNPs from different coding regions was considerably lower (Figure 3). There was no LD among associated sites between color pattern intervals. Finer examination revealed a complex haplotype structure, where different sets of individuals had genotypes associated with a color pattern phenotype at each of the associated SNPs, resulting from several recombination events between the different associated sites. As a result, there was no obvious haplotype structure that could explain color pattern phenotype.

### Expression analysis of candidate genes

**Figure 5:**
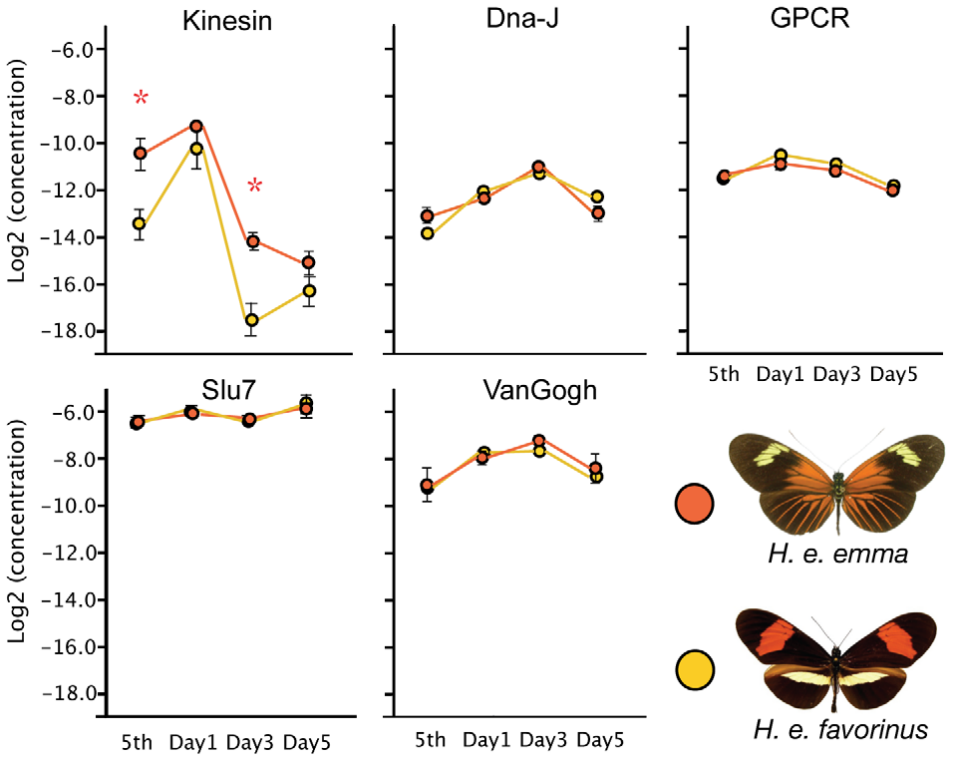
Quantitative PCR of *D*-interval candidate genes implicates *kinesin*. Quantitative PCR data for five candidate genes in the *D*-interval. Y-axis values are Log_2_ transformed values of the initial concentration of the gene divided by the EF-1α initial concentration; developmental stage is displayed on the X axis, including 5^th^ instar larvae and pupal developmental days 1, 3, and 5. Bars represent standard error among the biological replicates.

None of the SNPs in this study had a fixed association with color pattern, suggesting that, while the site is strongly associated with color pattern, they are not the functional variants themselves. However, the obvious implication is that they are near the functional site, which could be in *cis*-regulatory regions that act by causing differences in gene expression. To test this possibility, we compared overall transcription levels between the two races during the early stages of wing development (5^th^ larval instar and 1, 3, and 5 days after pupation), on genes at the *D* locus that had SNPs strongly associated with wing pattern phenotype either in *H. erato* or *H. melpomene* [33]. All genes, with the exception of *Slu7*, showed significant differences in expression across wing developmental stages (ANOVA: p<0.0001 to 0.0066; Bayesian Model Averaging: Pr(β ≠ 0) = 100 for each gene) (Figure 5). *Kinesin*, however, was the only candidate gene to show significant differences in expression between *H. e. emma* and *H. e. favorinus* (overall race effect p = 0.0001). Expression of this gene was roughly 8× higher in *H. e. emma* in 5^th^ instar larvae (p = 0.0028, t-test) and three days after pupation (p = 0.0014, t-test), than in *H. e. favorinus*. As with the ANOVA, statistical testing using Bayesian Model Averaging assigned strong probabilities to racial differences only with *Kinesin* (Pr(β≠0) >92.5), although a small race effect is predicted for *GPCR* (Pr(β≠0) >54.7; higher in *H. e. favorinus*).

## Discussion

The genomic regions that underlie pattern variation in *Heliconius* are “hotspots” of phenotypic evolution [13]. They underlie adaptive variation among races and species with both *convergent* and *highly divergent* wing patterns [29]–[31] and play an important role in speciation [16]–[18]. This study, together with the companion study [33], provides the first descriptions of the patterns of nucleotide diversity, LD, and gene expression across these evolutionary important genomic intervals. Our data highlight a complex history of recombination and gene flow across a sharp phenotypic boundary in *H. erato* that both reshapes our ideas about molecular basis of phenotypic change and focuses future research on a small set of candidate genes that are likely responsible for phenotypic variation in this extraordinary adaptive radiation.

### No molecular signature of recent, strong selection on color patterns

The genetic patterns that we observed are inconsistent with the evolution of novel wing patterns in *H. erato* via a very recent strong selective sweep on a new mutation or recent genetic bottleneck as have been proposed [41]. A selective sweep on a new adaptive variant, which quickly fixes beneficial alleles, is expected to generate a temporary genomic signature marked by a reduction of nucleotide variation and an increase in LD around selected sites as a result of genetic hitchhiking [42]. Empirically, these patterns have been observed around loci important in domestication (e.g. rice [43] and dogs [44],[45]), plant cultivation (sunflowers [46] and maize [47]), drug resistance (*Plasmodium*, [48]), and the colonization of new environments in the last 10,000 years (sticklebacks, [49]–[51]). In all cases, selection has been strong, directional, and very recent.

The genetic patterns across regions responsible for phenotypic variation in *H. erato* and *H. melpomene* serves as a cautionary note and may be more typical of the functional variation found in nature. In *H. erato*, per locus selection coefficients are high [34],[35]; yet, we see neither a strong reduction in genetic diversity nor extended LD across color pattern intervals. There are loci with nucleotide diversity patterns that deviate significantly from the neutral expectations, but not in a manner consistent with a recent, strong selective sweep acting on a new mutation. In all three loci in the *D* interval with the strongest association with color pattern, the patterns of nucleotide variation were largely consistent with neutrality (Table 1). Thus, recombination has essentially reduced the signature of selection to very narrow regions tightly linked to the sites controlling the adaptive color pattern variation. This pattern is consistent with the hypothesis that pattern diversification in *H. erato* is quite ancient, dating perhaps into the Pliocene (see [27]). Interestingly, we see a very similar pattern in *H. melpomene*, which likely radiated much more recently [27]. Alternatively, the patterns in both *H. erato* and *H. melpomene* could also be the result of a recent “soft sweep”, where selection acts on pre-existing variation [52],[53]. Thus, the allelic variants modulating particular color pattern elements are themselves old but the combination of patterning loci that characterize specific wing pattern phenotypes might have evolved much more recently [54],[55]. Under either scenario, however, the observed patterns in both *H. erato* and *H. melpomene* highlight the extent with which recombination can erase the signature of strong selection in natural populations [56].

The rapid decay of LD across both *H. erato* color pattern intervals marks a history of considerable recombination. Narrow hybrid zones between differently adapted populations are common in nature [32]. Hybrid individuals are frequently less fit than parental genotypes and these zones are typically envisioned as “population sinks” that are maintained by the movements of individuals from outside [32],[57],[58]. As a result, hybrid zones tend to show LD even among unlinked loci [59]–[62]. Instead of a population sink, the narrow transition zone between *H. e. favorinus* and *H. e. emma* can be more appropriately viewed as a population sieve- where population sizes have remained large, where recombination breaks down associations among alleles even at tightly linked loci, and gene flow allows most of the genome to be freely exchanged between the divergent races. Mallet observed similarly low population differentiation across this cline at 14 unlinked allozyme loci (average *F_ST_* = 0.038, unpublished data). Indeed, there is very little evidence for extended LD around loci that are responsible for adaptive differences in wing pattern and only slight genetic divergence between *H. e. emma* and *H. e. favorinus* at most of 25 coding regions examined within the two color pattern intervals (Figure 4). The only exceptions are regions tightly linked to the sites controlling the color variation, and, even here, LD decays rapidly with physical distance and estimates of *F_ST_* become moderate, albeit higher than at unlinked loci (see Table 2 and Table S2). The decay in LD in *H. erato* occurs faster than in *H. melpomene*, where there is evidence for strong LD (*r^2^*>>0.5) extending hundreds of kilobases across the *B* and *Yb* color pattern intervals [33]. Nonetheless, in both co-mimics, LD decays much more rapidly than has been reported near adaptive loci in sympatric host races of the pea aphid, *Acrythosiphon pisum* [63] and sympatric populations of lake whitefish, *Coregonus sp.* [64]. In the pea aphid study in particular, Via & West [63] showed that strong LD and strong genetic differentiation around the genomic intervals that underlie variation in ecologically important traits extends tens of centimorgans, presumably representing several Mbp at least. This is probably due to lower rates of cross-mating and geneflow, coupled with the largely non-recombinogenic reproduction in the pea aphid throughout most of the year. Our results are more similar to those found between *M* and *S* forms of *Anopheles gambiae*, where a few tens of genes around the centromeres and telomeres are the only regions with strong divergence [65]. Although in this case, the evolutionary or ecological forces driving these differences are largely unknown.

### The power of association mapping—localizing candidate regions underlying phenotypic variation

The observation that LD in *H. erato* populations around ecologically important traits decays at a rate more similar to *Drosophila* than pea aphids or mammals [63], [66]–[68] has important practical ramifications. Foremost, it means that naturally occurring *Heliconius* hybrid zones can be used to localize genomic regions responsible for adaptive differences in wing coloration at an extremely fine scale. On average there are informative polymorphic sites (with a minor allele frequency greater than 5%) every 30 bp within coding regions in our data on *H. erato*. Given this, along with the observed pattern of LD, surveying polymorphism every 200–500 bp should be sufficient to capture haplotype structure across the Peruvian hybrid zone and to finely localize genomic regions responsible for pattern variation in *H. erato*.

Even with our current coarse sampling, we were able to greatly narrow the candidate *D* and *Cr* intervals and focus research on a small set of candidate genes. Across the *D* interval, there are strong genotype-by-phenotype associations and high levels of genetic differentiation between phenotypically pure populations in three dispersed coding regions: *Dna-J*, *GPCR*, and *VanGogh*. Although several genes near these association peaks have strong sequence similarity to *Drosophila* genes with known molecular or biological functions that make them candidates for color pattern genes, only one, *kinesin*, showed strong expression differences between *H. e. emma* and *H. e. favorinus* (Figure 5) during early wing development. Kinesin proteins are known to play a role in pattern specification at a cellular level in *Drosophila* [69] and are involved in vertebrate [70] and invertebrate pigmentation [71]. We expect patterning loci to act as “switches” between different morphological trajectories and for the genes involved to show distinctive spatial/temporal shifts in expression patterns similar to what we observed in *kinesin*. Although future expression and functional validation is needed, we observed similar expression shifts in the *H. melpomene kinesin* [33], which further implicates this gene as playing a causal role in pattern variation in *Heliconius* and provides convincing evidence that parallel changes in gene regulation underlies the independent origins of these co-mimetic lineages.

Across the *Cr* interval, the two coding regions with the strongest associations, consist of a gene with strong homology to the *Drosophila* gene *Unkempt*, and another predicted gene with a leucine-rich repeat (*LRR*). These loci are approximately 80 kb apart. The *H. erato Unkempt* codes for the type of protein potentially involved in pattern generation. It contains a zinc-finger binding motif and is potentially a signaling molecule that can regulate the downstream expression of other genes. Indeed, the *Drosophila Unkempt* is involved in a number of developmental processes including wing and bristle morphogenesis [72]. The role of *Unkempt* in bristle morphogenesis is particularly intriguing, as the overlapping scales that color a butterfly wing are thought to have evolved from wing bristles [73]. Moreover, the different color scales in *Heliconius* have unique morphologies and are pigmented at different times during wing development [74], suggesting that pattern variation may be tied directly to scale maturation. If *Unkempt* is shown by additional research to be modulating pattern variation, it could provide yet another example of a conserved developmental gene co-opted to produce novel variation [75]–[77]. Alternatively, it may turn out that the gene that underlies pattern variation in *Heliconius* is either Lepidoptera-specific or has diverged significantly in both form and function from other insect lineages. *LRR* has no strong ortholog in *Drosophila*, the honeybee (*Apis mellifera*), or the flour beetle (*Tribolium castaneum*). It is most similar to the *Drosophila* gene, *Sur-8*, which is inferred to have RAS GTPase binding activity. This suggests it may be involved in signal transduction. This gene also showed the highest differentiation among *H. melpomene* races and between *H. melpomene* and *H. cydno* [33], further implicating this gene and the surrounding regions.

Three unlinked genomic intervals, *D*, *Cr* and *Sd*, interact to generate the phenotypic differences between *H. e. favorinus* and *H. e. emma* [40]. Yet, the overall effect on phenotype of variation across each of these loci is not identical and the much lower levels of population differentiation in the *Cr* interval relative to the *D* interval is likely due a combination of differences in dominance and selection on the two loci. In *H. erato*, there is a strong dominance hierarchy among the colored scale cells, where red scale cells (containing xanthommatin and dihydro-xanthommatin) are dominant to black (containing melanin) scale cells and both are dominant to yellow (containing 3-hydroxy-L-kynurenine) scale cells. For *H. e. emma* and *H. e. favorinus* this means that the *D* locus is effectively codominant, whereas the *Cr* allele for the *emma* lack of hindwing bar is dominant to presence of yellow hindwing bar in *favorinus* [40]. Additionally, the analysis of cline width, together with the overall percentage of wing surface affected suggests that the selection on the codominant *D* locus is much higher (*s*≈0.33) than selection on the dominant *Cr* locus (*s*≈0.15) [23],[34]. Thus, the power of natural selection to remove poorly adapted alleles at *Cr* is reduced, especially on the *H. e. emma* side of the zone where the recessive yellow bar alleles are rare [34]. In *H. melpomene* the *Yb* and *B* locus are homologous to the *H. erato Cr* and *D* loci, respectively, and are under similar selective constraints at this Peruvian hybrid zone. Similarly, a reduction in the power of natural selection on the *Cr* would likely result in a similar reduction of selection on *Yb*, which may explain why genetic differentiation between the *H. melpomene* Peruvian races is, like *H. erato*, much lower at genes near the *Yb* relative to the *B* locus (see [33], Table 1). Given the history of recombination implied by our data, we expect only sites extraordinarily tightly linked to the causative polymorphisms to yield strong associations. Collectively the association results across the *D* and *Cr* intervals highlight the power of using these naturally occurring hybrid zones to select candidate loci for future focused studies. Similar and possibly independently derived transitions between “postman” and “rayed” races of *H. erato* and *H. melpomene* that occur in Bolivia, Ecuador, Colombia, Suriname, and French Guiana, provide additional replicates to test the repeatability of evolution [19]–[21],[78],[79].

### The locus of evolution

The color pattern genes of *Heliconius* are classical examples of large effect loci where allelic variation causes major phenotypic shifts in the distribution of melanin and ommochrome pigments across large sections of both the fore- and hindwing. We are accustomed to thinking of the generation of phenotypic variation in terms of single causal mutations. This paradigm has shaped our ideas about how variation is produced by DNA sequences, and, although consistent with some of the handful of examples [2],[5],[80], there are reasons to imagine this is not the whole story, or even a dominant trend [75],[77],[81],[82].

In this light, the varying pattern of LD across the *D* and *Cr* intervals and the observation that different polymorphic sites are associated with pattern phenotype in different sets of individuals seems inconsistent with a single causal functional site. Our coarse sampling provides only a preliminary snapshot of haplotype structure across these intervals and genetic hitching, drift and ancestry can create complex genotype-by-phenotype signatures [83]–[86]. Nonetheless, given the rapid breakdown of LD observed, we would expect to see a much narrower window of association if variation was explained by a single causal site. However, the pattern we observe is expected if there are multiple functional sites dispersed across these intervals. Although LD was generally higher, a similar pattern was evident in *H. melpomene* [33]. These emerging genetic signatures are consistent with early ideas that these patterning loci were “supergenes” composed of a cluster of tightly linked loci [21]. For example, in *H. erato* the *D* locus was originally described as three unique loci, *D*, *R*, and *Y* [21] and there has been one *H. erato* individual collected in the Peruvian hybrid zone which had a DR/y recombinant phenotype [40]. In *H. melpomene* both the *B* and *Yb* loci, have roughly equivalent phenotypic effects as the *D* and *Cr* loci in *H. erato*, and have been clearly shown to be parts of tightly linked clusters of loci that control the end wing pattern phenotype. It is possible that these “clusters” are a series of enhancer elements that influence target gene expression and the terminal phenotype in an overall switch-like fashion [87]. Selection to maintain these clusters may explain the reduced recombination rate we observed across color pattern intervals in the pedigree-based linkage mapping of the *D* and *Cr* intervals and the large haplotype blocks across the *Yb* and *B* intervals in the Peruvian *H. melpomene* races [33]. However, in *H. erato* thousands of generations of hybridization in the middle of the hybrid zone may have allowed recombination to break apart functional sites, creating the mosaic of different haplotypes we observed across these intervals. Collectively, these two companion studies serve as natural replicates of how convergence on a similar adaptive trait can be independently derived and provide compelling evidence that similar genetic changes can underlie the evolution of Müllerian mimicry.

Our initial sampling of genetic variation across the color pattern loci provides important insights into the complex haplotype structure that potentially underlies the major phenotypic shifts in wing color patterns. These data suggest that finer genetic dissection of these hybrid zones and other parallel transitions will allow direct tests of the number and type of changes that underlie adaptive color pattern variation in *Heliconius*. These studies will help pinpoint functionally important polymorphisms and determine if a single functional site or multiple sites underlies adaptive color pattern variation and if these sites are changes in coding regions, in *cis*-regulatory regions, or both. Ultimately, linking the genetic changes underlying phenotypic variation with the development pathways involved in patterning the wing promises a whole new understanding of how morphological variation is created through development and modified by natural selection within the context of an adaptive radiation.

## Methods

We generated several F_2_ and backcross mapping families by crossing four different geographic races of *H. erato* to the same stock of *H. himera*. All crosses were carried out in the *Heliconius* insectaries at the University of Puerto Rico from stocks originally collected in the wild under permit from the Ministerio del Ambiente in Ecuador. We followed segregating variation at the *D* locus in the crosses involving *H. e. cyrbia*, *H. e. erato*, *H. e. etylus* and *H. e. notabilis*. We were also able to follow segregating variation for the *Cr* locus in crosses between *H. himera* and *H. e. cyrbia*. After eclosion, individuals were euthanized, had their wings removed for later morphological analysis, and their bodies were stored in DMSO at −80°C.

Genomic DNA was extracted from 1/3 of preserved thoracic tissue for each individual using Plant DNeasy Tissue kit (Qiagen Inc). Across the *D* interval, segregating variation was followed for one microsattelite and seven gene-based markers and for seven gene based markers across the *Cr* interval. The markers *D23/24* and *GerTra* were screened using the methods described in [29],[88]. All other gene-based markers were PCR amplified, the PCR product was purified Using ExoSAP-IT (USB), and sequenced in both directions using Big Dye Terminator v3.1 and run on a 3700 DNA Sequence Analyzer (Applied BioSystems). Due to an indel polymorphism at Gn47 some samples could be sequenced in the forward direction for Gn47. To overcome this, individuals identified as recombinants at Gn47 (see below) were PCR amplified and sequenced twice to confirm individual genotypes. For gene-based markers, primers were designed from available *H. erato* and/or *H. melpomene* BAC sequences, Table S1 provides the primer sequences, PCR conditions, and marker types. Sites and/or insertion/deletions that varied between the parents of a mapping family were screened among the offspring of those mapping families. To determine the distance between each marker and a color pattern locus, we looked for evidence of recombination between the genotype at each marker and the wing pattern phenotype. The greater the number of individuals showing a recombination event between a marker genotype and the color pattern phenotype, the further that specific marker was from the functional site(s) controlling the color pattern variation. For the *D* locus, the markers *Gn12*, *VanGogh* and *THAP* were only screened for a ‘recombinant panel’ of individuals. The recombinant panel consisted of all individuals identified as recombinants at markers *Gn47* and *D23/24*, six individuals from each mapping that were not recombinants at *Gn47* and *D23/24* and the parents of each mapping family. This method dramatically reduced the number of individuals screened and allowed us to efficiently determine the number of single recombination events between each marker and the *D* locus.

### Targeted *BAC* sequencing

We screened the *H. erato* BAC libraries, to identify BAC clones that spanned the *D* and *Cr* color pattern intervals. For the *Cr* locus, probes were designed from the previously published *H. erato* BAC clone 38A20 (AC193804) and *H. melpomene* BAC clones 11J7 (CU367882), 7G12, (CT955980) and 41C10 (CR974474) [29],[88]. Across the *D* locus, probes were designed from the *H. erato* BAC clone 25K04 (AC216670) and *H. melpomene* BAC clones 7G5 (CU462858) 27I5 (CU467807), and 28L23 (CU467808) that have been previously shown to be located near the *D* locus [31]. BAC library probing, fingerprinting of positively identified clones, and the sequencing and assembly of BAC clones that most extended our tiling coverage was done using the methods described in [88]. BAC clone sequences were aligned using SLAGAN [89] to create contiguous *H. erato* consensus sequences across the *D* and *Cr* color pattern loci. SLAGAN was also used to align these *H. erato* consensus sequences with available *H. melpomene* BAC sequences and *Bombyx mori* genome sequence to confirm the order, orientation and locations of gaps among the *H. erato* sequences. The consensus *H. erato* sequences were then annotated using Kaikogaas (http://kaikogaas.dna.affrc.go.jp), an automated annotation package that implements several gene prediction methods to identify potential coding regions. Locations of predicted coding regions and conserved domains are shown in Figure S2. Primers for probes were designed from potential coding regions using the methods described above, in *Butterfly Crosses and Fine-Mapping*. Primer sequences and PCR conditions for probes are available in Table S1.

### Population sampling

Individuals used in this study were collected from five locations transecting 32 km across a *H. erato* hybrid zone in Eastern Peru near Tarapoto. In total we sampled 76 individuals, 20 from phenotypically “pure” populations of *H. e. favorinus* in Tarapoto and Rio Pansillo, 14 individuals from a primarily phenotypically “pure” population of *H. e. emma* in Davidcillo, and 42 from admixed populations in Pongo de Cainarache and Barranquitas located near the center of the hybrid zone. Due to dominance and strong epistasis between the three loci, when individuals have a *D_emma_D_emma_* genotype, the *Cr_emma_Cr_emma_* and *Cr_emma_Cr_fav_* genotypes are phenotypically indistinguishable. Therefore some individuals were assigned a *Cr_emma_-* dominant genotype (see [34]), indicating the genotype of the second *Cr* allele is unknown. Individual's names, sex, race, color pattern genotype and collection location are recorded in Table S4.

### Genomic sampling

Nucleotide variation was sampled across two candidate intervals controlling major changes in warning color patterns, as well as three other autosomal genes unlinked to color pattern. We sampled twelve coding regions from *D* interval, ten from the *Cr* interval, and three coding regions from genes on unlinked chromosomes (Table S1). Names of coding regions are based on sequence homology to annotated genes in other organisms, or if no sequence homology was found numbered gene names were assigned. On average, 520 bp fragments were sampled every 47 kb across a candidate color pattern interval. Primer design for PCR amplification and sequencing was done using Primer3 [90]. Primers for the three unlinked loci were developed by Pringle *et al.* [91], and have been shown to map to different linkage groups in *H. melpomene*. Primer sequences and PCR conditions for each locus can be found in Table S1. Genomic DNA extraction, PCR product purification and sequencing were completed using the same methods as described above. For some individuals, *Abhydrolase* was cloned from purified PCR product using TOPO cloning kit (Invitrogen) and 4–10 clones were sequenced. Ambiguous bases in the genomic sequences were cleaned and trimmed manually using Sequencher (Gene Codes Corporation). A site was determined to be heterozygous if the lower quality nucleotide had a peak height at least 50% of the higher quality nucleotide. Sequences were initially aligned using Sequencher and the resulting alignments were then manually adjusted.

### Genetic diversity analyses

Population genetic estimates of nucleotide diversity, population differentiation and tests of neutrality were conducted using SITES and HKA [92]. Nucleotide diversity was estimated as *π* (average number of pair-wise differences per base pair) for all samples i) within the *H. e. favorinus* population ii) within the *H. e. emma* population and iii) within the admixed population. This was done for each of the 25 sampled coding regions independently and by concatenating all coding regions sampled on the same chromosome (Table S1). Tajima's *D* [93] was also calculated for all coding regions independently and by concatenating them, to examine for departures from the neutral model of evolution. For each coding region 10,000 coalescent simulations based on locus specific estimates of theta were used to determine if the observed patterns of nucleotide diversity and locus specific estimates of Tajima's *D* significantly departed from neutral expectations using the program HKA as described in [94]. *F_ST_* was estimated between the two phenotypically pure populations and the admixed population for each coding region and using SITES (Table 2) and FDIST2 (Table S2) [95].

### Linkage disequilibrium

To determine the extent of LD across the candidate color pattern intervals in *Heliconius*, we computed composite LD estimates for 432 SNPs from the 25 coding regions we sampled. Of the 1542 polymorphic sites identified in this study, 442 sites had a minor allele frequency greater than 0.05 and were considered informative for LD analyses. Multi-allelic sites that had a minor allele with a frequency less than 0.05 were condensed to bi-allelic SNPs by merging the minor allele genotypes. Ten polymorphic sites had 2 or 3 minor alleles with a frequency greater than or equal to 0.05 that were not condensed to bi-allelic SNPs and were not included in the LD analyses. LD between the remaining 432 SNPs was executed using the commonly used composite estimate of LD method described by Weir [96], which does not assume HWE or that haplotypes are known . LD among the 432 SNPs was estimated independently for i) all samples ii) within the *H. e. favorinus* population iii) within the *H. e. emma* population and iv) within the admixed population. LD between the 432 SNPs using all sampled individuals was visualized with GOLD [97], by plotting the composite *r^2^* estimates between all pair wise SNP combinations. To visualize the difference in mean *r^2^* between the three populations, a sliding window average of *r^2^* across 50 neighboring SNPs was calculated independently for each population and plotted by distance.

### Genotype-by-phenotype association

We determined if any SNP was associated with a color pattern phenotype using chi-squared linear trend test [96]. This test assumes a linear relationship between the phenotype and genotype and applies a chi-square goodness-of-fit test to determine if the genotype at a SNP is significantly associated with a particular wing color pattern. For the association tests we used bi-allelic and multi-allelic SNPs with minor allele frequencies equal to or greater than 0.05. Color pattern phenotypes at the *D* and *Cr* loci were scored as 0.0 representing *H. e. favorinus* phenotypes, 0.5 representing hybrid phenotypes and 1.0 *H. e. emma* phenotypes. Individuals with *H. e. emma D* phenotypes *and H. e. emma Cr* phenotypes were assigned 1.0 for the *D* phenotype score and 0.5 for the *Cr* phenotype score, due to the effects of dominance previously mentioned; varying the *Cr* value for from 0.5 to 1.0 for these individuals had a negligible effect on the association test results (data not shown).

### Quantitative examination of gene expression

We used quantitative PCR involving SYBR Green technology to detect transcript levels of *kinesin*, *Slu7*, *GPCR*, *Dna-J*, and *VanGogh* in butterfly forewing tissues. Samples of whole forewings were dissected from December, 2008 - February, 2009 from reared *H. e. emma* and *H. e. favorinus* stocks founded from multiple individuals collected within 30 km of one another in Peru. We staged individuals indoors at 25°C starting in early 5^th^ larval instar. Chosen larval wings were at mid-5^th^ instar, stage 2.25–2.75 based on the work of Reed and colleagues [98]. Pupal stages were based on the time after the pupal molting event, including Day 1 (24hrs), Day 3 (72hrs), and Day 5 (120hrs). We sampled three individuals of each stage and race, resulting in 2 races × 4 stages × 3 biological replicates = 24 specimens. All specimens were processed randomly from dissection through amplification stages.

We extracted total RNA from the tissues using an electric tissue homogenizer and the RNAqueous Total RNA Isolation Kit (Ambion). This procedure was followed by a TURBO DNA-free (Applied Biosystems) treatment to remove genomic DNA contaminants. Extracted products were run through the Agilent Bioanalyzer to ensure the RNA was of high quality. For cDNA systhesis, 0.4 µg of each sample was added to the standard 20µl reaction procedure outlined in the High-Capacity cDNA Reverse Transcription Kit (Applied Biosystems). Resulting products were diluted with an additional 50µl.

For each gene, we performed quantitative PCR on all 24 samples in triplicate to correct for technical error. We used EF-1α as a standard to normalize the expression of the test genes. Primers for amplification of cDNA were designed using recommended criteria and range from 98 – 175 bp in length (see Table S3). We ran primer sets through an initial qPCR optimization to test for optimal primer concentrations and ran DNA-free controls to test for primer-dimers. qPCR reactions were run using 1µl of 5µM primers (0.5µl for GPCR), 5µl SYBR Green Mix, 1 µl template, and water to 10 µl. Reactions were run in 384-well plates in the Applied Biosystems 7900HT Fast Real-Time PCR machine under standard mode and absolute quantification for 40 cycles of 95°C for 15 sec, 60°C for 60 sec. Each cycle was followed by a dissociation step to measure the dissociation temperature of the sample, which tests for primer-dimer and differences in sequences among samples. A standard curve was generated for each gene using a 10^−3^ to 10^−7^ dilution series drawn from a PCR amplified product using the same primers.

To normalize Ct values from the qPCR run, we first calculated the mean of each of the three technical replicates. We then calculated initial concentrations for each sample for each gene given the equation of the standard curve for that gene. These initial concentrations were divided by the inferred concentration of EF-1α for that sample, thus correcting for inconsistencies in initial cDNA sample concentrations. These relative values were then log_2_ transformed for presentation and analysis. Log_2_ transformation is necessary to normalize the variances of the samples and represents expression differences in more biologically realistic fold differences. Significance values were obtained from a two-way ANOVA using stage, race, and race*stage as effects. Effects of race within each stage were further dissected for each gene using series of t-tests and an FDR of 0.05 (threshold at p = 0.0028) to correct for multiple testing. In addition to a general ANOVA and to compare our results to the companion paper [33], we used a combination of generalized linear regression models (GLMs) and Bayesian Model Averaging (BMA) on the non-log transformed data to model the effect of race, developmental stage, and their interactions, on gene expression. These statistics were performed using the ‘bic.glm’ function in the ‘BMA’ package [99] implemented in R (R Development Core Team 2008).

## Supporting Information

Figure S1 LD decays rapidly with distance in *Heliconius erato*. Composite LD estimates between SNPs within the same coding region across both color pattern intervals and unlinked loci. Dashed vertical line at 550 bp, designates the average size of coding regions sampled and demonstrates that LD decays rapidly within the genes.
(0.24 MB TIF)

Figure S2 BAC annotations of *D* and *Cr* loci. Annotations of BAC sequences using Kaikogaas. Approximate locations of predicted coding regions are shown along BAC sequences of the and *Cr* intervals. Coding regions sampled for this study are colored red and the annotation has a double box. All other predicted coding regions are shown in green and the annotation has only a single box. For predicted coding regions with significant similarity to protein sequences in GenBank using blastp, the accession number and organism name for the sequence with highest similarity is given. Conserved domains identified in the PFAM database are also shown. In general, gene content and order is largely preserved between *H. erato* and *H. melpomene* across the *D* interval (see [33]). For a more detailed annotation of the homologous genomic regions in *H. melpomene*, see [33] and [101].
(1.99 MB PDF)

Table S1 Key locus information including gene tag/number, location, PCR primers, and accession numbers.
(0.06 MB PDF)

Table S2 Summary of association results and *F_ST_* estimates for each SNP.
(1.06 MB TXT)

Table S3 qPCR primer information.
(0.04 MB PDF)

Table S4 Key sample information, including race, collection location and color pattern genotype.
(0.04 MB PDF)
