# Supplementary figures and images for: Genomic Hotspots for Adaptation: The Population Genetics of Müllerian Mimicry in Heliconius erato
Source: PLoS Genet. 2010 Feb 5;6(2):e1000796. doi: 10.1371/journal.pgen.1000796 (PMC2816678; doi:10.1371/journal.pgen.1000796)

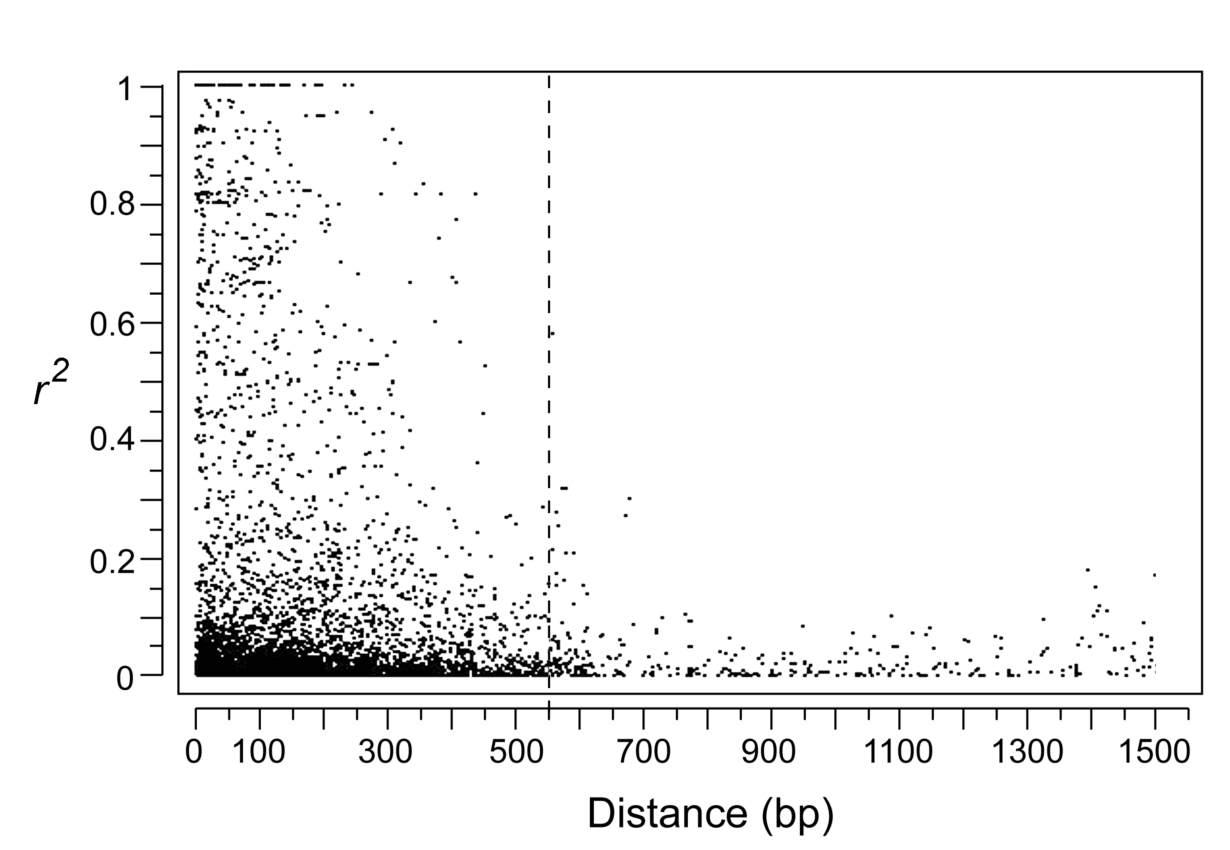

Supplement: Figure S1 — LD decays rapidly with distance in Heliconius erato. Composite LD estimates between SNPs within the same coding region across both color pattern intervals and unlinked loci. Dashed vertical line at 550 bp, designates the average size of coding regions sampled and demonstrates that LD decays rapidly within the genes. (0.24 MB TIF) [file pgen.1000796.s001.tif]

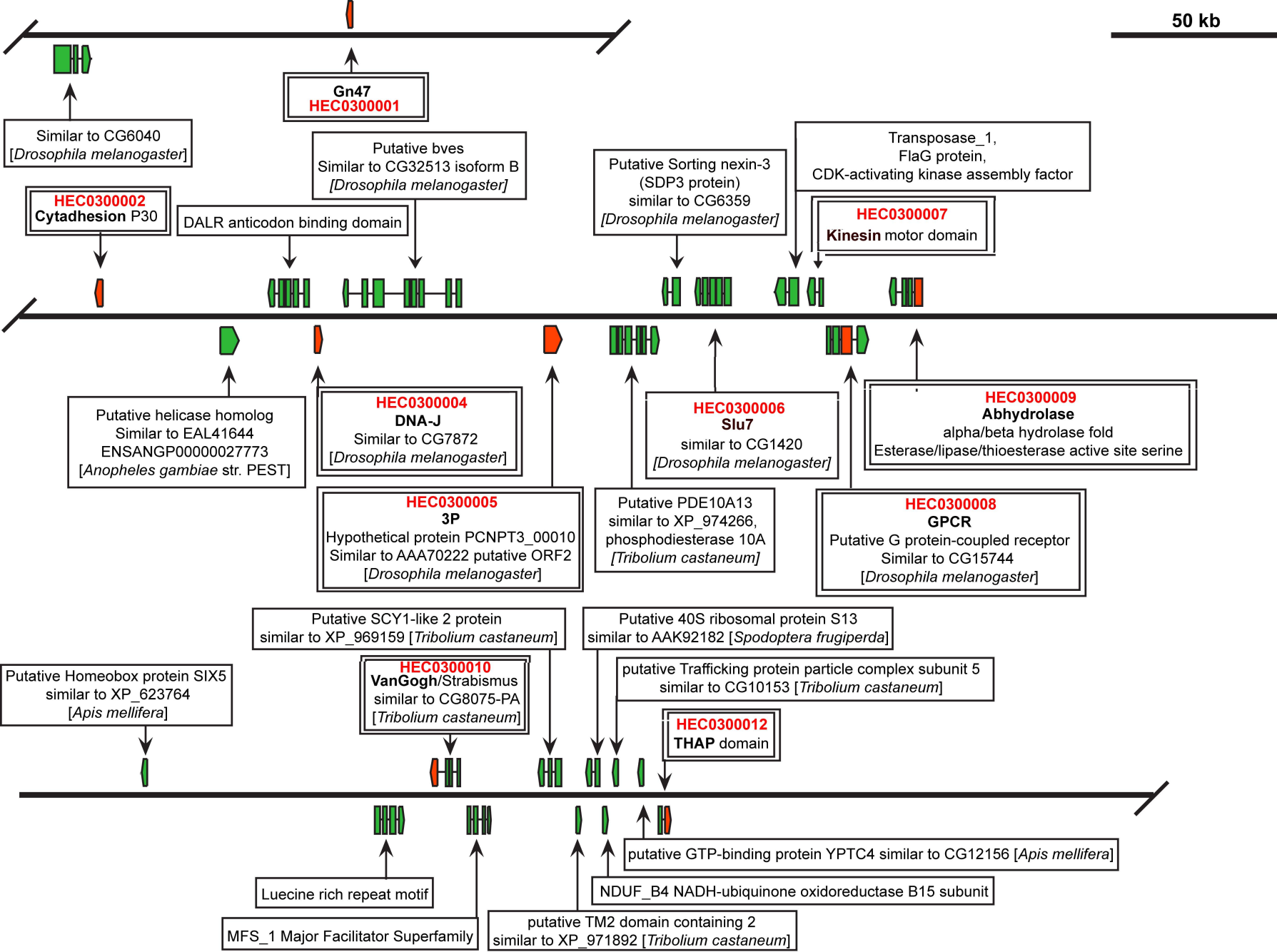

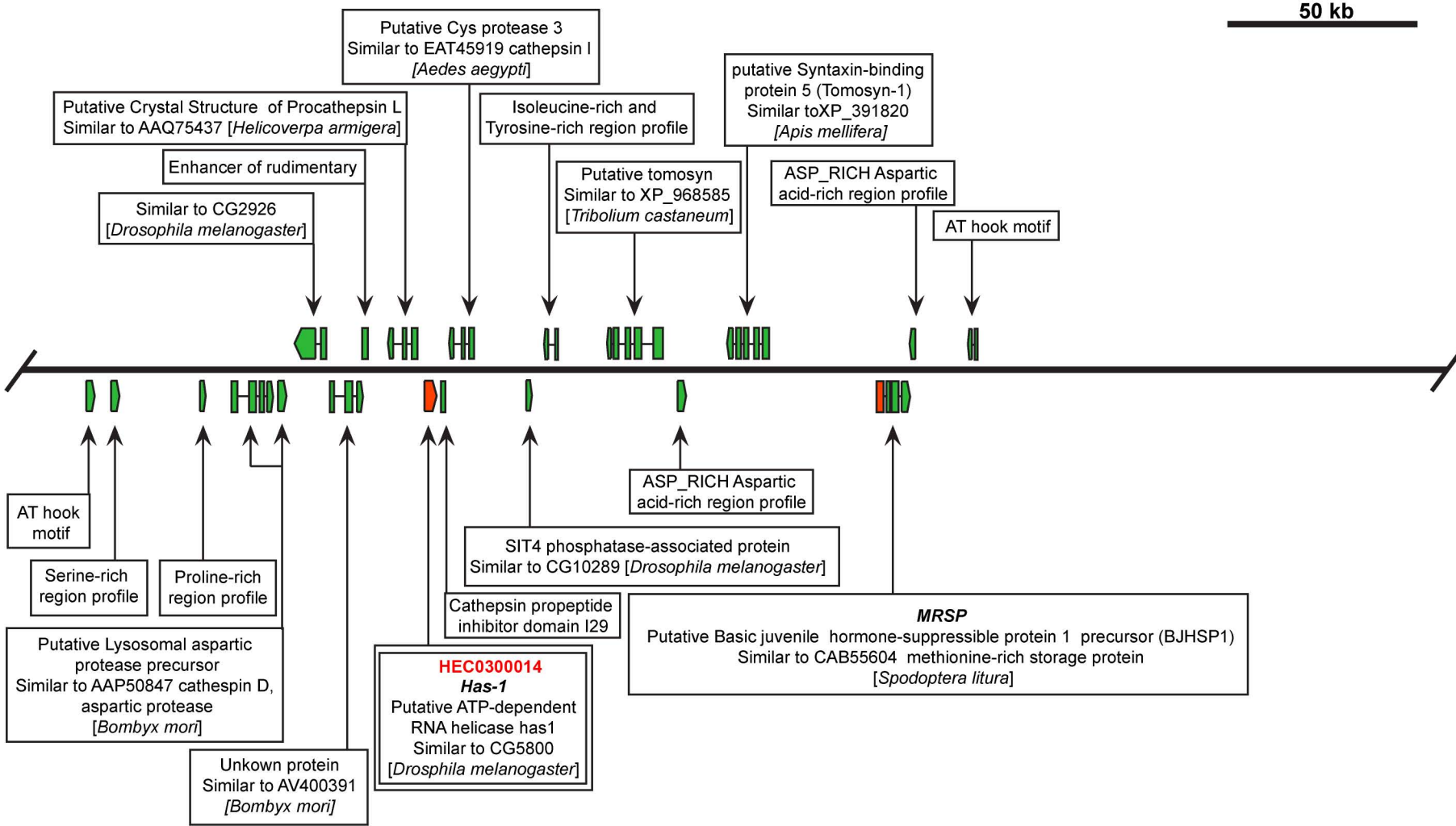

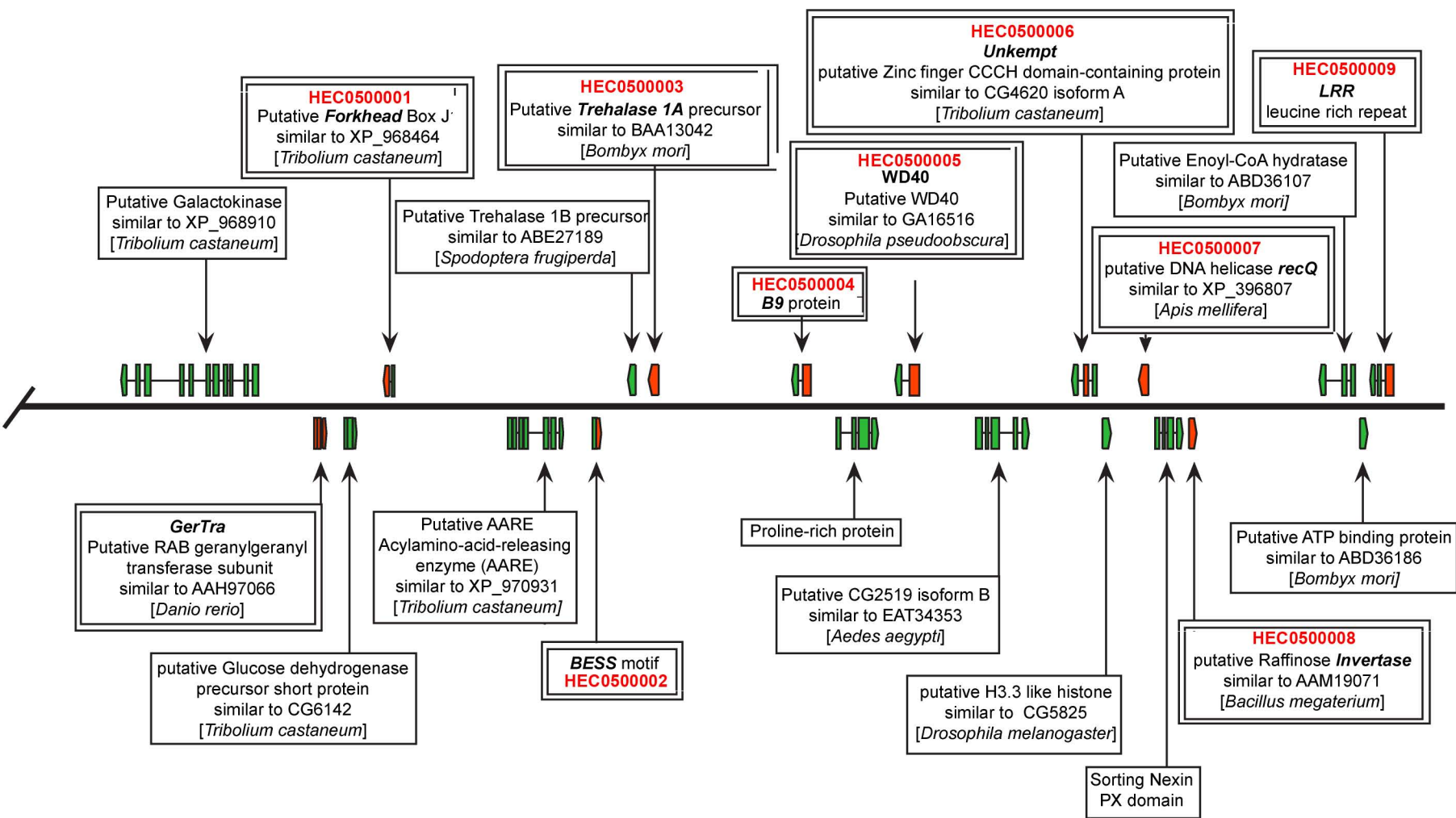

Supplement: Figure S2 — BAC annotations of D and Cr loci. Annotations of BAC sequences using Kaikogaas. Approximate locations of predicted coding regions are shown along BAC sequences of the and Cr intervals. Coding regions sampled for this study are colored red and the annotation has a double box. All other predicted coding regions are shown in green and the annotation has only a single box. For predicted coding regions with significant similarity to protein sequences in GenBank using blastp, the accession number and organism name for the sequence with highest similarity is given. Conserved domains identified in the PFAM database are also shown. In general, gene content and order is largely preserved between H. erato and H. melpomene across the D interval (see [33]). For a more detailed annotation of the homologous genomic regions in H. melpomene, see [33] and [101]. (1.99 MB PDF) [file pgen.1000796.s002.pdf]
