# Supplementary material for: Genomic Hotspots for Adaptation: The Population Genetics of Müllerian Mimicry in Heliconius erato
Source: PLoS Genet. 2010 Feb 5;6(2):e1000796. doi: 10.1371/journal.pgen.1000796 (PMC2816678; doi:10.1371/journal.pgen.1000796)
Supplement: Table S1 — Key locus information including gene tag/number, location, PCR primers, and accession numbers. (0.06 MB PDF) [file pgen.1000796.s003.pdf]

Locus tag	HE gene number	HM gene number	LG[A]	Brood[B]	Pop[C]	Probe[D]	Pos[E]	Primer	Intron	GB Acc.G
Gn47	HEC0300001	-	HEC03	yes	no	yes	1	GACGCGTGAAGAAATTCAATTCCG<br>GTACAACACGCGACTGTTGTGAG	yes	in submission
Cytadhesion	HEC0300002	-	HEC03	no	yes	yes	91,726	GGCCTATACCCAAACGGTTGAG<br>AGCGTAGTACCAATACCCAATG	no	in submission
Gn12	HEC0300003	-	HEC03	yes	no	no	109,416	GGATTGTCAGTCGCAAGTCA<br>GGATTTCGCGGTGAACTAAG	yes	in submission
Dna-J	HEC0300004	HM01006	HEC03	yes	yes	yes	165,009	GTGCTCGGTGTCACAAGAGA<br>CTTCCAAAGCCATCTTGCAT	no	in submission
3P	HEC0300005	-	HEC03	no	yes	no	237,736	GCCATTTCCTATCTGCATACC<br>CAACCATCGCTTTTAGTC	yes	in submission
Slu7-exon9	HEC0300006	HM01019	HEC03	no	yes	no	283,611	AAGCTCTGAAAGCAGTTCCGAC<br>TTAACATGCCTTCTTCAGTTTG	no	in submission
Slu7-exon2	HEC0300006	HM01019	HEC03	no	yes	no	287,640	GGAGCAAAATTCACAAGTGCAG<br>TCTTGGCTCTCAATTCCCT	no	in submission
kinesin	HEC0300007	HM01018	HEC03	no	yes	no	299,335	TTTATTGAACCTAGGCCACC<br>AGTTAAGTCTATCAGCACCTCT	no	in submission
GPCR	HEC0300008	HM01017	HEC03	yes	yes	yes	309,248	GTTACACATGCCCGGTGATAA<br>CGTCTCTCAGCCTCATTG	no	in submission
Abhydrolase	HEC0300009	-	HEC03	no	yes	yes	322,422	GCCCATAAATCAACTGTTTCTG<br>GGTTTGTGGCAAGTAATCC	yes	in submission
VanGogh	HEC0300010	HM01038	HEC03	yes	yes	yes	513,028	TAGCTTGTGCCTTCCTCTGAGA<br>GGGATGGGCAAGAAGTTA	no	in submission
Gene 6	HEC0300011	-	HEC03	no	yes	yes	517,433	AAGACTTGTACGAATGGCACCT<br>AAGCTCCGAAATCAGAAGA	no	in submission
THAP	HEC0300012	-	HEC03	yes	yes	yes	552,580	TACCAAAGCAATGATGAACG<br>AGCTCAACGACACTTTACAGGA	no	in submission
Gene 18	HEC0300013	-	HEC03	no	yes	no	579,264	AACGACCGAGCACCTACCATTA<br>GTCTTCTAAAAGTCCCATCGC	no	in submission
Has1	HEC0300014	-	HEC03	yes	yes	yes	1,442,402	CTAGAAGCCAAATCTGTCGCAAAT<br>TTCAGGTCGTGCCACATGA	no	in submission
D23/24	-	-	HEC03	yes	no	no	1,751,244	AGTACTAAGTCTGAGCACGTGTGA<br>CCATTCTAAGCACCGATAACA	NA	in submission
Forkhead	HEC0500001		HEC05	no	yes	no	1	TTCGCCTAAATTAAATAAAACGGTA<br>TCATCATGCACATAACATCAGG	no	in submission
BESS	HEC0500002	-	HEC05	no	yes	no	54,672	GAACTCCGACAGCGTTCAG<br>ATTCTACGGAGCCCTCCAC	no	in submission
Trehalase (A)	HEC0500003	HM00004	HEC05	no	yes	no	66,809	TGTACTCTCCACCACCACCA	no	in submission

								AGATTACCCTGGCGGTATCC		
Trehalase (B)	HEC0500003	HM00004	HEC05	no	yes	no	67,583	CGGTCCTGCTGGTTCTATTC<br>TGCCATATTTTGAAGAGCATTT	no	in submission
B9	HEC0500004	HM00007	HEC05	yes	yes	yes	104,621	CGGGAATCTGAAGTAACAAA<br>ACTAGCTGAGGCCCTATTAAA	no	in submission
WD40	HEC0500005	HM00010	HEC05	no	yes	no	130,877	ATTATAAGCAGCTATCCACGC<br>AAAGCAGCCGACCAAC	no	in submission
Unkempt	HEC0500006	HM00013	HEC05	no	yes	no	177,423	CTGCAGCGGCGTCTGACAGTAG<br>CACCGTGCTGCAAGAGGTC	no	in submission
RecQ	HEC0500007	HM00017	HEC05	yes	yes	no	190,761	TGCTACAGCTCATGTTCTGTCTG<br>CCCTTTTGTCTGAATGGAACTGGT	no	in submission
Invertase	HEC0500008	-	HEC05	yes	yes	no	201,977	CCCATCTATCACATAACCGCTCCT<br>CCGAGCCACGCGATAAGAA	no	in submission
LRR	HEC0500009	HM00024	HEC05	yes	yes	yes	249,391	CGTGAAGTACCGACTGTTGTAC<br>CATAATTTCTCAGGGAGCATACAT	no	in submission
Gn71	HEC0500010	-	HEC05	yes	no	no	292,390	TATTGCGAGTTGAGCTGTGG<br>ATGCGCCATTTCCAGTTAAG	yes	in submission
HEAT	HEC0500011	-	HEC05	yes	no	no	~500000	CACTTTCGGAAACGGATCAGT<br>CGCGGTGCACTCGAACAATAT	no	in submission
RNU3	HEC0500012	-	HEC05	yes	no	no	~650000	CGCTCCTAACCTCGAAAATG<br>GTGCTCCTGCCCTATAGCTG	no	in submission
Caspase	-	-	HEM20	no	yes	no	NA	AATGCCTGTGGAAAGAAACG<br>ACCAGAGGGTGTCAGGCTTA	no	in submission
SUZ12	-	-	HEM07	no	yes	no	NA	ACGAGTTCACGGATGTCA<br>ATATGGAGGACCGTTTGC	no	in submission
Wingless	-	-	HEM01	no	yes	no	NA	CCCAGTTTTAGATCTGTCG<br>TCTCGGTCCGGTATCCGCG	no	in submission

A = linkage group for each marker from [33-35]; B = locus used in pedigree-based linkage mapping; C = locus used for population sampling; D = locus used for BAC library probing; E = approximate location of locus along BAC tile path; F = forward and reverse primers used for PCR amplificaiton and sequencing; G = Genbanck accession numbers.
