# Supplementary material for: Genomic Hotspots for Adaptation: The Population Genetics of Müllerian Mimicry in Heliconius erato
Source: PLoS Genet. 2010 Feb 5;6(2):e1000796. doi: 10.1371/journal.pgen.1000796 (PMC2816678; doi:10.1371/journal.pgen.1000796)
Supplement: Table S3 — qPCR primer information. (0.04 MB PDF) [file pgen.1000796.s005.pdf]

Gene	Primer Name	Primer Sequence	Amplified size (bp)	Intron size (bp)
DnaJ	DNAJ_HH_F2	GTGCTCGGTGTCACAAGAGA	142	none
	DNAJ_HH_R3	CGGTGGCGATACGATTAAAG		
GPCR	GPCR_HH_F	GAGGGCACAGAGCTAAGGAT	126	589
	GPCR_HH_R	TCTAGGAGTTGACCGTGCAT		
kinesin	kinesin_HH_F7	TTGGGACATACAGCCTTTCG	171	814
	kinesin_HH_R7	CGCCTGAGAAGAATGACAATG		
Slu7	Slu7_HH_E1MOD_F1	CGTGGAGCCATGACTCATAA	98	773
	Slu7_HH_E1E2_R1	TTGAATTCATCAGGAGCTATGC		
VanGogh	VanGogh_HH_F4	CGCTCGCTAGAGCCTTACAA	130	454
	VanGogh_HH_R4	GAGATGCGTCTTGGGACAGA		
EF-1a	EF1a_HH_F5	TGCCAGTAGGCAGAGTTGAA	175	none
	EF1a_HH_R5	AATTCCTTGACGGACACGTT		
