# Supplementary material for: Genomic Hotspots for Adaptation: The Population Genetics of Müllerian Mimicry in Heliconius erato
Source: PLoS Genet. 2010 Feb 5;6(2):e1000796. doi: 10.1371/journal.pgen.1000796 (PMC2816678; doi:10.1371/journal.pgen.1000796)
Supplement: Table S4 — Key sample information, including race, collection location and color pattern genotype. (0.04 MB PDF) [file pgen.1000796.s006.pdf]

ID	Genus, species, race	D	Cr	Sd	Collection location
JM0853	*Heliconius erato favorinus*	*dd*	*crcr*	*sdsd*	Tarapoto, Peru
JM1511	*Heliconius erato favorinus*	*dd*	*crcr*	*sdsd*	Tarapoto, Peru
JM1513	*Heliconius erato favorinus*	*dd*	*crcr*	*sdsd*	Tarapoto, Peru
JM1514	*Heliconius erato favorinus*	*dd*	*crcr*	*sdsd*	Tarapoto, Peru
JM1515	*Heliconius erato favorinus*	*dd*	*crcr*	*sdsd*	Tarapoto, Peru
JM1277	*Heliconius erato favorinus*	*dd*	*crcr*	*sdsd*	Tarapoto, Peru
JM1003	*Heliconius erato favorinus*	*dd*	*crcr*	*sdsd*	Rio Pansillo, Peru
JM1004	*Heliconius erato favorinus*	*dd*	*crcr*	*sdsd*	Rio Pansillo, Peru
JM1005	*Heliconius erato favorinus*	*dd*	*crcr*	*sdsd*	Rio Pansillo, Peru
JM1011	*Heliconius erato favorinus*	*dd*	*crcr*	*sdsd*	Rio Pansillo, Peru
JM1298	*Heliconius erato favorinus*	*dd*	*crcr*	*sdsd*	Rio Pansillo, Peru
JM1008	*Heliconius erato favorinus*	*dd*	*crcr*	*sdsd*	Rio Pansillo, Peru
JM1009	*Heliconius erato favorinus*	*dd*	*crcr*	*sdsd*	Rio Pansillo, Peru
JM1010	*Heliconius erato favorinus*	*dd*	*crcr*	*sdsd*	Rio Pansillo, Peru
JM1289	*Heliconius erato favorinus*	*dd*	*crcr*	*sdsd*	Rio Pansillo, Peru
JM0996	*Heliconius erato favorinus*	*dd*	*crcr*	*sdsd*	Rio Pansillo, Peru
JM0997	*Heliconius erato favorinus*	*dd*	*crcr*	*sdsd*	Rio Pansillo, Peru
JM1001	*Heliconius erato favorinus*	*dd*	*crcr*	*sdsd*	Rio Pansillo, Peru
JM1013	*Heliconius erato favorinus*	*dd*	*crcr*	*sdsd*	Rio Pansillo, Peru
JM1364	*Heliconius erato favorinus x emma*	*DD*	*Cr -*	*Sdsd*	Pongo de Cainarache , Peru
JM1365	*Heliconius erato favorinus x emma*	*dd*	*Cr -*	*Sdsd*	Pongo de Cainarache , Peru
JM1367	*Heliconius erato favorinus x emma*	*Dd*	*crcr*	*Sd -*	Pongo de Cainarache , Peru
JM1368	*Heliconius erato favorinus x emma*	*DD*	*Crcr*	*Sdsd*	Pongo de Cainarache , Peru
JM1369	*Heliconius erato favorinus x emma*	*dd*	*Crcr*	*sdsd*	Pongo de Cainarache , Peru
JM1370	*Heliconius erato favorinus x emma*	*DD*	*Crcr*	*Sd -*	Pongo de Cainarache , Peru
JM1371	*Heliconius erato favorinus x emma*	*dd*	*crcr*	*Sdsd*	Pongo de Cainarache , Peru
JM1372	*Heliconius erato emma*	*DD*	*Cr -*	*Sd -*	Pongo de Cainarache , Peru
JM1373	*Heliconius erato favorinus x emma*	*Dd*	*Crcr*	*Sdsd*	Pongo de Cainarache , Peru
JM1375	*Heliconius erato favorinus x emma*	*DD*	*crcr*	*Sd -*	Pongo de Cainarache , Peru
JM1376	*Heliconius erato favorinus x emma*	*Dd*	*crcr*	*Sd -*	Pongo de Cainarache , Peru
JM1377	*Heliconius erato emma*	*DD*	*Cr -*	*Sd -*	Pongo de Cainarache , Peru
JM1381	*Heliconius erato favorinus x emma*	*DD*	*Cr -*	*Sdsd*	Pongo de Cainarache , Peru
JM1382	*Heliconius erato favorinus x emma*	*DD*	*crcr*	*Sd -*	Pongo de Cainarache , Peru
JM1383	*Heliconius erato favorinus x emma*	*Dd*	*Crcr*	*sdsd*	Pongo de Cainarache , Peru
JM1384	*Heliconius erato favorinus x emma*	*Dd*	*Cr -*	*Sdsd*	Pongo de Cainarache , Peru
JM1386	*Heliconius erato favorinus x emma*	*Dd*	*Crcr*	*sdsd*	Pongo de Cainarache , Peru
JM1411	*Heliconius erato favorinus x emma*	*dd*	*Cr -*	*Sdsd*	Pongo de Cainarache , Peru
JM1412	*Heliconius erato favorinus x emma*	*Dd*	*Cr -*	*Sdsd*	Pongo de Cainarache , Peru
JM1413	*Heliconius erato favorinus x emma*	*DD*	*Crcr*	*sdsd*	Pongo de Cainarache , Peru
JM1642	*Heliconius erato favorinus x emma*	*dd*	*crcr*	*Sdsd*	Pongo de Cainarache , Peru
JM1643	*Heliconius erato favorinus x emma*	*Dd*	*Cr -*	*Sdsd*	Pongo de Cainarache , Peru
JM1625	*Heliconius erato emma*	*DD*	*Cr -*	*Sd -*	Barranquitas, Peru
JM1627	*Heliconius erato favorinus x emma*	*DD*	*Cr -*	*Sdsd*	Barranquitas, Peru
JM1628	*Heliconius erato emma*	*DD*	*Cr -*	*Sd -*	Barranquitas, Peru
JM1629	*Heliconius erato favorinus x emma*	*Dd*	*Cr -*	*Sd -*	Barranquitas, Peru
JM1631	*Heliconius erato favorinus x emma*	*Dd*	*Cr -*	*Sd -*	Barranquitas, Peru
JM1632	*Heliconius erato favorinus x emma*	*DD*	*Crcr*	*Sd -*	Barranquitas, Peru
JM1634	*Heliconius erato emma*	*DD*	*Cr -*	*Sd -*	Barranquitas, Peru
JM1639	*Heliconius erato favorinus x emma*	*DD*	*Cr -*	*Sdsd*	Barranquitas, Peru
JM1641	*Heliconius erato favorinus x emma*	*Dd*	*Cr -*	*Sdsd*	Barranquitas, Peru
JM1694	*Heliconius erato favorinus x emma*	*DD*	*Cr -*	*Sdsd*	Barranquitas, Peru
JM1695	*Heliconius erato emma*	*DD*	*Cr -*	*Sd -*	Barranquitas, Peru
JM1697	*Heliconius erato emma*	*DD*	*Cr -*	*Sd -*	Barranquitas, Peru

JM1699	*Heliconius erato favorinus x emma*	*Dd*	*Cr -*	*Sdsd*	Barranquitas, Peru
JM1700	*Heliconius erato emma*	*DD*	*Cr -*	*Sd -*	Barranquitas, Peru
JM1701	*Heliconius erato favorinus x emma*	*DD*	*Cr -*	*Sdsd*	Barranquitas, Peru
JM1704	*Heliconius erato favorinus x emma*	*Dd*	*Cr -*	*Sdsd*	Barranquitas, Peru
JM1706	*Heliconius erato favorinus x emma*	*Dd*	*Cr -*	*Sdsd*	Barranquitas, Peru
JM1707	*Heliconius erato favorinus x emma*	*DD*	*Cr -*	*Sdsd*	Barranquitas, Peru
JM1708	*Heliconius erato favorinus x emma*	*dd*	*Cr -*	*Sdsd*	Barranquitas, Peru
JM1712	*Heliconius erato emma*	*DD*	*Cr -*	*Sd -*	Barranquitas, Peru
JM1028	*Heliconius erato favorinus x emma*	*Dd*	*Cr -*	*sdsd*	Davidcillo, Peru
JM1029	*Heliconius erato emma*	*DD*	*Cr -*	*Sd -*	Davidcillo, Peru
JM1031	*Heliconius erato emma*	*DD*	*Cr -*	*Sd -*	Davidcillo, Peru
JM1033	*Heliconius erato emma*	*DD*	*Cr -*	*Sd -*	Davidcillo, Peru
JM1034	*Heliconius erato emma*	*DD*	*Cr -*	*Sd -*	Davidcillo, Peru
JM1574	*Heliconius erato emma*	*DD*	*Cr -*	*Sd -*	Davidcillo, Peru
JM1575	*Heliconius erato favorinus x emma*	*DD*	*Crcr*	*Sdsd*	Davidcillo, Peru
JM1576	*Heliconius erato emma*	*DD*	*Cr -*	*Sd -*	Davidcillo, Peru
JM1577	*Heliconius erato favorinus x emma*	*Dd*	*Cr -*	*Sd -*	Davidcillo, Peru
JM1578	*Heliconius erato emma*	*DD*	*Cr -*	*Sd -*	Davidcillo, Peru
JM1579	*Heliconius erato favorinus x emma*	*DD*	*Cr -*	*Sdsd*	Davidcillo, Peru
JM1581	*Heliconius erato emma*	*DD*	*Cr -*	*Sd -*	Davidcillo, Peru
JM1582	*Heliconius erato favorinus x emma*	*DD*	*Crcr*	*Sd -*	Davidcillo, Peru
JM1589	*Heliconius erato favorinus x emma*	*DD*	*Cr -*	*Sd -*	Davidcillo, Peru
